# Carbon Fiber Reinforced Polymers

**DOI:** 10.3390/ma14195545

**Published:** 2021-09-24

**Authors:** Francesca Lionetto

**Affiliations:** Department of Engineering for Innovation, University of Salento, Via Monteroni, 73100 Lecce, Italy; francesca.lionetto@unisalento.it

The current demand for lightweight and high-performance structures leads to increasing applications of carbon fiber reinforced polymers, which is also made possible by novel production methods, automation with repeatable quality, the reduced cost of carbon fibers, out of autoclave processes such as resin transfer molding and resin infusion technologies, the re-use of waste fibers, development in preform technology, high-performance, fast-curing resins, etc. [1,2]. Moreover, the diffusion of a multi-material design, where metallic and non-metallic materials are used together to fabricate the same component, has driven the research towards efficient joining technologies of metals to carbon fiber reinforced composites. More recently, the introduction of nanofillers into conventional carbon fiber reinforced polymers offered the opportunity to combine the potential benefits of nanoscale reinforcement with well-established fibrous composites to create multiscale or hierarchical composites, characterized by enhanced structural and functional properties.

The Special Issue *Carbon Fiber Reinforced Polymers* collects 31 papers, 3 reviews and 28 research papers, with the aim to present recent advances in carbon fiber reinforced polymers, focusing on emerging trends in matrix development, composite manufacturing technologies and joining methods and mechanical behavior modeling. 

Concerning advances in polymer matrix research, Wang et al. [3] analyzed a low-cost and fast-cure epoxy resin cured with an imidazole hardener, suitable for the liquid molding of automotive composite parts. The preparation of nanostructured matrices has attracted great interest, as proved by the work of Barra et al. [4], who compared different industrial mixing methods for dispersing multi-walled carbon nanotubes into an aeronautical epoxy resin, also evaluating the properties of the resulting carbon fiber reinforced laminate by vibroacoustic performance and flammability. Dimoka et al. [5] investigated the influence of multi-walled carbon nanotubes on the improvement of damage tolerance after impact of Out of Autoclave (OoA) carbon fiber reinforced polymer laminates. 

Concerning the development of new technologies, Sun et al. [6] prepared high-performance composites by combining traditional Resin Transfer Molding and compression molding, obtaining composites with superior mechanical properties and reduced porosity. Xie et al. [7] analyzed the effects of different process parameters (i.e., compression temperature, compression pressure, pressure-holding time and cooling rate) on the mechanical performance of composites. In order to increase the impact resistance, interlaminar strength and integrity of conventionally laminated composites, 3D textile composites were invented. Zhu et al. [8] studied the bending properties of zigzag-shaped 3D woven spacer composites with different directions, different numbers of weaving cycles and different heights by experiments and FEM simulation.

Advances in additive manufacturing technologies and composite materials are starting to be combined with synergic procedures to achieve personalized and high-performance solutions. Munoz-Guijosa et al. [9] demonstrated the benefits of 3D-printed rapid molds, upon which composite fibers can be laminated in a direct and resource-efficient way, for the personalized development of articular splints. The rapid mold concept has allowed for a flexible lamination and curing process, even compatible with autoclaves. Donadei et al. [10] investigated the causes of delamination and porosities during press forming of pre-consolidated flat laminates (blanks) made of carbon fiber reinforced poly(ether ketone ketone). They demonstrated that deconsolidation phenomena can be related to residual stresses formed during blank forming in an autoclave, then released during infrared heating of the blank, determining most of the defects. Xu et al. [11] investigated the tribo-drilling of high-strength CFRP composites by addressing the aspects of frictional heat and drilling temperatures at varying cutting conditions. They accounted for the elastic recovery effect during the drilling process.

Challenges in composite manufacturing can only be faced with a correct process design. Lionetto et al. [12] presented an experimentally validated model to simulate the resin infusion process of an aeronautical component by accounting for the chemo-physical and rheological changes in the crosslinking resin and the measurement of the in-plane and out-of-plane permeability of the reinforcement with an in-house-built ultrasonic set-up [13]. The numerical simulation of the resin infusion process involved modeling of the resin flow through the reinforcement, heat exchange in the part and within the mold and the crosslinking reaction of the resin.

Defect detection in a composite component can be used for understanding and predicting its mechanical behavior. This is of great importance in the aeronautical field because the irregularities of the composite material could compromise functionality. Casavola et al. [14] analyzed T-pull CFRP samples by means of non-destructive testing and evaluated their effect on the mechanical response of the material. This study represented a first step in developing a new procedure for the quick and easy inspection of CFRP T-joints.

Wang et al. [15] investigated the interlaminar shear behavior of CFRP specimens by microscale strain mapping. A three-point bending device was developed under a laser scanning microscope, and the full-field strain distributions, including normal, shear and principal strains on the cross section of CFRP, in a three-point bending test, were measured using a developed sampling Moiré technique. Ma et al. [16] investigated the effects of different defect typologies on the bending properties of CFRP composites, determining the main causes of defects and the control measures to avoid defects. Hosoi et al. [17] proposed a model for the prediction of fatigue life for transverse crack initiation of CFRP cross-ply and quasi-isotropic laminates.

In civil engineering, externally bonded fiber-reinforced polymers (FRPs) have been shown to be an effective strengthening method for existing engineering structures due to their excellent tensile strength, light weight, corrosion resistance and easy tailoring. The performance depends on adequate bond strength between the FRP and the concrete, which ensures the stress transmission. Liang et al. [18] performed a reliability analysis to study the bond behavior of the FRP–concrete interface under wet–dry cycles and sustained loading. They demonstrated the use of probabilistic modeling to evaluate long-term performance based on a series of double-lap pullout test results. Min et al. [19] studied the debonding initiating and propagating process along the FRP–concrete interface under fatigue load and proposed a new prediction model. In particular, they focused on intermediate crack-induced debonding near the loading point, achieving an excellent agreement between model and experimental results. Li et al. [20] investigated the bond–slip relationship for CFRP sheets externally bonded to concrete under cyclic loading, proposing a bilinear bond–slip model which considered the effects of the cyclic load level, concrete strength, and CFRP-to-concrete ratio. Since the early detection of debonding between CFRP and concrete is of crucial importance, Liu et al. [21] investigated the feasibility of using low-cost piezoceramic sensors to detect and monitor the debonding of CFRP-reinforced concrete beams in situ.

Kaeseberg et al. [22] presented a constructive and critical assessment of the design methodologies available for CFRP-confined RC columns. Significant dissimilarities among codes and guidelines of different countries were found, leading to different and mostly contrary results. Some new findings concerning the rupture strength and the maximum strength plus accompanying axial strain of a CFRP-confined column are suitable to improve the current guidelines. Ding et al. [23] prepared chemically bonded phosphate ceramic (CBPC) fiber-reinforced composites at indoor temperatures by using an aluminum phosphate binder. Their applications can fill the gap between traditional hydraulic cements and sintered ceramics. 

Lambiase et al. [24] reviewed the state-of-the-art on advanced joining processes for metal–composite and metal–polymer hybrid structures, which are now are in high demand in several fields. They discussed the main limitations of conventional joining processes and described the joining mechanisms, the main differences, advantages, and limitations of new joining processes. Three reference clusters were identified: fast mechanical joining processes, thermomechanical interlocking processes and thermomechanical joining processes. They proposed a new classification, providing a tool for orienting within the broad horizon of new joining processes for MMHSs. Liu et al. [25] optimized the bonded repair of cracked aluminum alloy plate by a microwave-cured sandwich composite patch. They firstly optimized the properties of the composite patches with different layer numbers and then evaluated their repair efficiency on a cracked aluminum alloy plate.

Several works have dealt with the modeling of mechanical behavior of composite materials. Zhang et al. [26] developed an in-house three-dimensional explicit finite element (FEM) model to investigate the dynamic responses of CFRPs under X-ray radiation in order to predict the dynamic behavior in a real service condition in spacecraft applications. The model represented a valid tool for protecting spacecraft from such unexpected damage. The CFRP composite was more effective than the aluminum panel in reducing radiation-induced pressure and BOI momentum. 

Dutra et al. [27] applied an expanded Puck and Schürmann Inter-Fiber Fracture criterion for predicting the failure envelopes of FRTP 3D-printed composite materials. The effect of the ratio between the transverse compressive strength and the in-plane shear strength was discussed and a new transition point between the fracture conditions under compressive loading was proposed. Wang et al. [28] critically reviewed the elastoplastic micromechanics models of composites and compared them in the case of the elastoplastic behavior of long fibrous composites subjected to a static load. Xie et al. [29] studied anisotropic viscoelastic constitutive models of compression molding for CFRP. 

Seong et al. [30] proposed an analytic scheme to predict deformations of a multi-layered fiber preform by comparing the forces applied to the preform in a mold. The model predicted the occurrence, type and position of fiber deformation, which agreed with the experimental results of the multi-layered preforms. Liu et al. [31] proposed an improved analytical solution to consider thermo-viscoelastic effects on residual stresses and deformations of flat composite laminates during curing. Furthermore, the mechanism generating residual stresses and deformations for unsymmetrical composite laminates was investigated based on the proposed analytical solution.

Koch et al. [32] evaluated and modeled the fatigue damage behavior of polymer composites at reversed cyclic loading. The mesoscopic damage initiation and progression in cross-ply laminates was analyzed to describe the material degradation during fatigue. Antin et al. [33] developed a multiscale modeling approach to estimate the effect of defects on the strength of unidirectional carbon fiber composites. The work encompassed a micromechanics approach and a 3D finite element model meshed directly from micrographs. The effect of porosity was simulated using a resin-rich area in the microstructure and the results were compared to experimental work on samples containing pores.

D’Mello et al. [34] investigated the influence of unit cell size and fiber packing on the transverse tensile response of fiber-reinforced composites in the context of integrated computational materials engineering. The results suggest that the choice of unit cell size was more sensitive to strength and less sensitive to stiffness when these properties were used as homogenized inputs to macro-scale models. 

The Guest Editor is confident that this Special Issue will benefit a broad community of academic and industrial researchers interested in advanced composites.

## References

[1] Dell’Anna R., Lionetto F., Montagna F., Maffezzoli A. (2018). Lay-Up and Consolidation of a Composite Pipe by In Situ Ultrasonic Welding of a Thermoplastic Matrix Composite Tape. Materials.

[2] Lionetto F., López-Muñoz R., Espinoza-González C., Mis-Fernández R., Rodríguez-Fernández O., Maffezzoli A. (2019). A Study on Exfoliation of Expanded Graphite Stacks in Candelilla Wax. Materials.

[3] Wang Y., Liu W., Qiu Y., Wei Y. (2018). A one-component, fast-cure, and economical epoxy resin system suitable for liquid molding of automotive composite parts. Materials.

[4] Barra G., Guadagno L., Vertuccio L., Simonet B., Santos B., Zarrelli M., Arena M., Viscardi M. (2019). Different Methods of Dispersing Carbon Nanotubes in Epoxy Resin and Initial Evaluation of the Obtained Nanocomposite as a Matrix of Carbon Fiber Reinforced Laminate in Terms of Vibroacoustic Performance and Flammability. Materials.

[5] Dimoka P., Psarras S., Kostagiannakopoulou C., Kostopoulos V. (2019). Assessing the Damage Tolerance of Out of Autoclave Manufactured Carbon Fibre Reinforced Polymers Modified with Multi-Walled Carbon Nanotubes. Materials.

[6] Sun Z., Xiao J., Tao L., Wei Y., Wang S., Zhang H., Zhu S., Yu M. (2019). Preparation of High-Performance Carbon Fiber-Reinforced Epoxy Composites by Compression Resin Transfer Molding. Materials.

[7] Xie J., Wang S., Cui Z., Wu J. (2019). Process optimization for compression molding of carbon fiber–reinforced thermosetting polymer. Materials.

[8] Zhu L., Lyu L., Zhang X., Wang Y., Guo J., Xiong X. (2019). Bending properties of zigzag-shaped 3D woven spacer composites: Experiment and FEM simulation. Materials.

[9] Munoz-Guijosa J.M., Zapata Martinez R., Martinez Cendrero A., Diaz Lantada A. (2020). Rapid prototyping of personalized articular orthoses by lamination of composite fibers upon 3D-printed molds. Materials.

[10] Donadei V., Lionetto F., Wielandt M., Offringa A., Maffezzoli A. (2018). Effects of Blank Quality on Press-Formed PEKK/Carbon Composite Parts. Materials.

[11] Xu J., Li C., Dang J., El Mansori M., Ren F. (2018). A study on drilling high-strength CFRP laminates: Frictional heat and cutting temperature. Materials.

[12] Lionetto F., Moscatello A., Totaro G., Raffone M., Maffezzoli A. (2020). Experimental and numerical study of vacuum resin infusion of stiffened carbon fiber reinforced panels. Materials.

[13] Lionetto F., Montagna F., Maffezzoli A. (2020). Out-Of-Plane Permeability Evaluation of Carbon Fiber Preforms by Ultrasonic Wave Propagation. Materials.

[14] Casavola C., Palano F., De Cillis F., Tati A., Terzi R., Luprano V. (2018). Analysis of CFRP Joints by Means of T-Pull Mechanical Test and Ultrasonic Defects Detection. Materials.

[15] Wang Q., Ri S., Tsuda H., Takashita Y., Kitamura R., Ogihara S. (2018). Interlaminar Shear Behavior of Laminated Carbon Fiber Reinforced Plastic from Microscale Strain Distributions Measured by Sampling Moiré Technique. Materials.

[16] Ma Y., Li S., Wang J., Ju L., Liu X. (2018). Influence of defects on bending properties of 2D-T700/E44 composites prepared by improved compression molding process. Materials.

[17] Hosoi A., Kawada H. (2018). Fatigue life prediction for transverse crack initiation of CFRP cross-ply and quasi-isotropic laminates. Materials.

[18] Liang H., Li S., Lu Y., Yang T. (2018). Reliability analysis of bond behaviour of CFRP–concrete interface under wet–dry cycles. Materials.

[19] Min X., Zhang J., Wang C., Song S., Yang D. (2020). Experimental investigation of fatigue debonding growth in FRP–concrete interface. Materials.

[20] Li K., Cao S., Yang Y., Zhu J. (2018). Bond–slip relationship for CFRP sheets externally bonded to concrete under cyclic loading. Materials.

[21] Liu S., Sun W., Jing H., Dong Z. (2019). Debonding Detection and Monitoring for CFRP Reinforced Concrete Beams Using Pizeoceramic Sensors. Materials.

[22] Kaeseberg S., Messerer D., Holschemacher K. (2019). Assessment of standards and codes dedicated to CFRP confinement of RC columns. Materials.

[23] Ding Z., Li Y.-Y., Lu C., Liu J. (2018). An investigation of fiber reinforced chemically bonded phosphate ceramic composites at room temperature. Materials.

[24] Lambiase F., Scipioni S.I., Lee C.-J., Ko D.-C., Liu F. (2021). A State-of-the-Art Review on Advanced Joining Processes for Metal-Composite and Metal-Polymer Hybrid Structures. Materials.

[25] Liu X., Wu J., Xi J., Yu Z. (2019). Bonded Repair Optimization of Cracked Aluminum Alloy Plate by Microwave Cured Carbon-Aramid Fiber/Epoxy Sandwich Composite Patch. Materials.

[26] Zhang K., Tang W., Fu K. (2018). Modeling of dynamic behavior of carbon fiber-reinforced polymer (CFRP) composite under X-ray radiation. Materials.

[27] Dutra T.A., Ferreira R.T.L., Resende H.B., Blinzler B.J., Larsson R. (2020). Expanding Puck and Schürmann inter fiber fracture criterion for fiber reinforced thermoplastic 3D-printed composite materials. Materials.

[28] Wang Y., Huang Z. (2018). Analytical micromechanics models for elastoplastic behavior of long fibrous composites: A critical review and comparative study. Materials.

[29] Xie J., Wang S., Cui Z., Wu J., Zhou X. (2020). Research on Anisotropic Viscoelastic Constitutive Model of Compression Molding for CFRP. Materials.

[30] Seong D.G., Kim S., Lee D., Yi J.W., Kim S.W., Kim S.Y. (2018). Prediction of Defect Formation during Resin Impregnation Process through a Multi-Layered Fiber Preform in Resin Transfer Molding by a Proposed Analytical Model. Materials.

[31] Zhang Y., Gao Z., Yang X., Chang J., Liu Z., Jiang K. (2019). Fish-scale-derived carbon dots as efficient fluorescent nanoprobes for detection of ferric ions. RSC Adv..

[32] Koch I., Just G., Brod M., Chen J., Doblies A., Dean A., Gude M., Rolfes R., Hopmann C., Fiedler B. (2019). Evaluation and modeling of the fatigue damage behavior of polymer composites at reversed cyclic loading. Materials.

[33] Antin K.-N., Laukkanen A., Andersson T., Smyl D., Vilaça P. (2019). A multiscale modelling approach for estimating the effect of defects in unidirectional carbon fiber reinforced polymer composites. Materials.

[34] D’Mello R.J., Waas A.M. (2019). Influence of unit cell size and fiber packing on the transverse tensile response of fiber reinforced composites. Materials.

